# mHealth Intervention for Dementia Prevention through lifestyle Optimisation (MIND-PRO) in a primary care setting: protocol for a randomised controlled trial in people with low SES and/or migration background

**DOI:** 10.1136/bmjopen-2024-088324

**Published:** 2025-02-03

**Authors:** Anne Roos van der Endt, Marieke P Hoevenaar-Blom, Henrike Galenkamp, Martien J H Kas, Esther van den Berg, Ron Handels, Eric P Moll van Charante*, Edo Richard*

**Affiliations:** 1 Department of Public and Occupational Health, Amsterdam UMC Location VUmc, Amsterdam, The Netherlands; 2 Donders Institute for Brain Cognition and Behaviour, Department of Neurology, Radboudumc, Nijmegen, The Netherlands; 3 Department of General Practice, Amsterdam UMC Location AMC, Amsterdam, The Netherlands; 4 Institute for Evolutionary Life Sciences, University of Groningen, Groningen, The Netherlands; 5 Department of Neurology and Alzheimer Center, Erasmus MC, Rotterdam, The Netherlands; 6 Alzheimer Centre Limburg, Department of Psychiatry and Neuropsychology, School of Mental Health and Neurosicences, Maastricht University, Maastricht, The Netherlands

**Keywords:** research design, cardiovascular disease, dementia, eHealth, risk management

## Abstract

**Introduction:**

The Mobile Health (mHealth) Intervention for Dementia Prevention through lifestyle Optimisation (MIND-PRO) study addresses the increasing prevalence of dementia among populations with lower socio-economic status (SES) and/or a migration background. The study aims to evaluate the effectiveness and implementation of an mHealth intervention designed for self-managing lifestyle modifications with remote coaching to reduce dementia risk factors.

**Methods and analysis:**

This prospective randomised open-label blinded end point (PROBE) trial follows a type 2 hybrid effectiveness-implementation design with a 12-month intervention period. It aims to recruit 692 participants in Dutch primary care. Entry criteria include age 50–75 years, low SES and/or migration background, one or more dementia risk factors (hypertension, dyslipidaemia, diabetes mellitus, physical inactivity, smoking, depression and overweight) or manifest cardiovascular disease and possession of a smartphone. Participants are randomised to a coach-supported, interactive app facilitating self-management of dementia risk factors or to a control app with static health information. The primary effectiveness outcome is a composite score of systolic blood pressure, non-high-density lipoprotein cholesterol and body mass index. Implementation outcomes include coverage, adoption, acceptability, appropriateness, feasibility, fidelity, costs and sustainability of the intervention. Secondary outcomes include the Cardiovascular Risk Factors, Ageing and Dementia risk score and its individual risk factors, and disability, physical activity, depressive symptoms, cognitive functioning and daily distance moved.

**Ethics and dissemination:**

The MIND-PRO trial is funded by the Netherlands Organisation for Health Research and Development (ZonMw, grant number 10510032120004) and approved by the Ethics Committee of Amsterdam UMC (reference: METC 2023.0770). Results are expected in 2026 and will be submitted for publication in a peer-reviewed journal, and presented at scientific conferences.

**Trial registration number:**

ISRCTN92928122.

**STRENGTHS AND LIMITATIONS OF THIS STUDY**

- Participants were selected to represent a high-risk, diverse group, including individuals with lower socio-economic status and migration backgrounds.
- This group is under-represented in conventional preventive initiatives, highlighting the importance of tailored, culture-sensitive interventions.
- In this proof-of-concept trial, both effectiveness and implementation of the intervention will be evaluated using a hybrid trial design.
- A potential limitation, common in preventive studies, is the risk of selection bias as participants might be healthier and more motivated to change their lifestyle.

## Introduction

### Background and rationale

 The global prevalence of dementia is expected to rise in the coming decades, which can primarily be attributed to population ageing and growth.^1^ Up to 40% of dementia is associated with modifiable risk factors, including high blood pressure, physical inactivity, unhealthy diet, overweight and smoking.^2^ These risk factors largely overlap with those of cardiovascular disease (CVD).^3^ Even a modest reduction of 10% in these risk factors can potentially have a major long-term impact on the prevalence of dementia due to the frequent occurrence of these risk factors, underlining the importance of preventive measures.^2^

The increase in dementia prevalence is not equally distributed worldwide. People living in low- and middle-income countries and people of lower socio-economic status (SES) and/or migration background, in high-income countries carry the highest burden.^1 4^ This could be attributed to the clustering convergence of risk factors within these groups,^5^ and restricted access to healthcare and prevention programmes.^6 7^ Within these high-risk groups, there is greater potential for improvement, yet they are frequently under-represented in prevention trials. The widespread use of mobile phones, coupled with increasing access to the internet via mobile devices, provides an opportunity for using Mobile Health (mHealth) technology to reach these underserved populations.^8^ It is crucial for developers of mHealth interventions and healthcare policymakers to recognise that these innovations have been primarily adopted by younger, highly educated individuals. This phenomenon inadvertently excludes people with lower (health) literacy skills, exacerbating the existing health inequality gap.^9 10^ In this study, we aim to mitigate this challenge by involving our target population in the process of developing the mHealth Intervention for Dementia Prevention through lifestyle Optimisation (MIND-PRO) intervention to ensure it is culture-sensitive and appropriate for those with low SES and/or a migration background.^11^ We will build on the lessons learnt from the Prevention of Dementia by Mobile Phone Applications (PRODEMOS)^12^,^14^ and the Healthy Aging through Internet Counseling of the Elderly (HATICE)^15^,^17^ trials to further improve a coach-supported smartphone app and tailor it to a population with a migration background and/or low SES facing elevated dementia risk in the Netherlands. The MIND-PRO trial aims to investigate the effectiveness and implementation of this culture-sensitive app supporting lifestyle modification to lower dementia risk factors through self-management and remote coaching during 12 months intervention.

## Methods

### Study design

The study is a single-centre, investigator-initiated, prospective, randomised, open-label blinded end point (PROBE) trial with 12 months intervention, conducted in the Netherlands. A type 2 hybrid implementation-effectiveness design will be used to assess proof of concept for effectiveness and implementation.^18^ The MIND-PRO trial is a project within the Netherlands Dementia Prevention Initiative (NDPI), part of the Dutch National Dementia Plan.

### Study population and recruitment

People aged between 50 and 75 years with a basic level of literacy in Dutch, of low SES and/or Turkish or South-Asian Surinamese origin, with one or more dementia risk factors or manifest CVD and in possession of a smartphone, are eligible for participation. Participants will be primarily recruited from the Healthy Life in an Urban Setting (HELIUS) study, a population-based observational prospective cohort within the largest migrant populations of Amsterdam, including the Dutch host population (n=24 781 included at baseline).^19^ By building on the HELIUS study infrastructure, we will be able to select and invite potential participants based on SES, ethnic origin and dementia risk factors, as these variables are available in the HELIUS database.^19^ Additional recruitment will be conducted through primary care practices if the target sample size cannot be achieved with HELIUS participants alone. Eligibility criteria for the RCT are summarised in box 1. Low SES is operationalised using self-reported educational attainment in the Netherlands or in the country of origin, specifically defined as (i) ‘none or only primary education’, (ii) ‘lower vocational or lower secondary education’, (iii) ‘intermediate vocational or intermediate or higher secondary education’ as previously collected in HELIUS.^19^ All participants will provide written informed consent (online supplemental appendix 2).

Box 1 **Overview of eligibility criteria**

**Exclusion criteria**

- Diagnosed with dementia by a specialist or general practitioner
- A score <21 on the Rowland Universal Dementia Assessment Scale^24^
- Any condition expected to limit 12 months compliance and follow-up
- Any impairment interfering with operation of a smartphone
- Participating in another randomised controlled trial on lifestyle behavioural change
- Present alcohol or illicit drug abuse impairing study participation

### Intervention and control condition

Participants randomised to the intervention arm will get access to an mHealth app aimed at lifestyle behavioural change, supported by a coach. This interactive app facilitates self-management of dementia risk factors (hypertension, dyslipidaemia, active smoking, overweight, lack of physical exercise and unhealthy diet). The participants will be supported by an experienced lifestyle coach trained in motivational interviewing, who will be matched to the ethnicity of the participants with one of the coaches able to speak Turkish for Turkish participants. The coaches will assist participants in formulating simple, measurable, achievable, realistic and time-bound (SMART) goals and support them in achieving these goals. Their role is to guide them in their lifestyle change. They are instructed to recommend participants with medical questions to seek help from their doctor. Also, mental health-related questions are beyond the scope of our intervention, and will result in a recommendation to visit their doctor. Participants can contact the coach via the app through chat, and coaches will be motivated to stay in contact with the participants at least once a month. After secure login, the app shows the participants’ dementia risk profile, based on baseline assessment. Participants can set goals for lifestyle change, monitor these goals and enter self-measurements, for example, weight, blood pressure and physical exercise. Progress over time is visually presented whenever feasible. Additionally, the app contains evidence-based education modules, including educational videos specifically designed for this study population, featuring the health coaches. The intervention app will be available in three versions for each ethnicity, with culturally acceptable content. The two versions for the Dutch and South-Asian Surinamese population will be available in Dutch; the version for the Turkish population will be available in both Dutch and Turkish. Those randomised to the control condition will have access to an app which is similar in appearance, but lacks the interactive features of coach-support and monitoring risk factors. Participants are informed that there are two versions of the app and that they will be randomly assigned to one. They are not made aware that one app serves as the active intervention while the other represents the control condition. The control app provides only static information about healthy lifestyles, without incorporating culturally specific content, and is available in both Dutch and Turkish.

### Patient and public involvement

**Figure 1:**
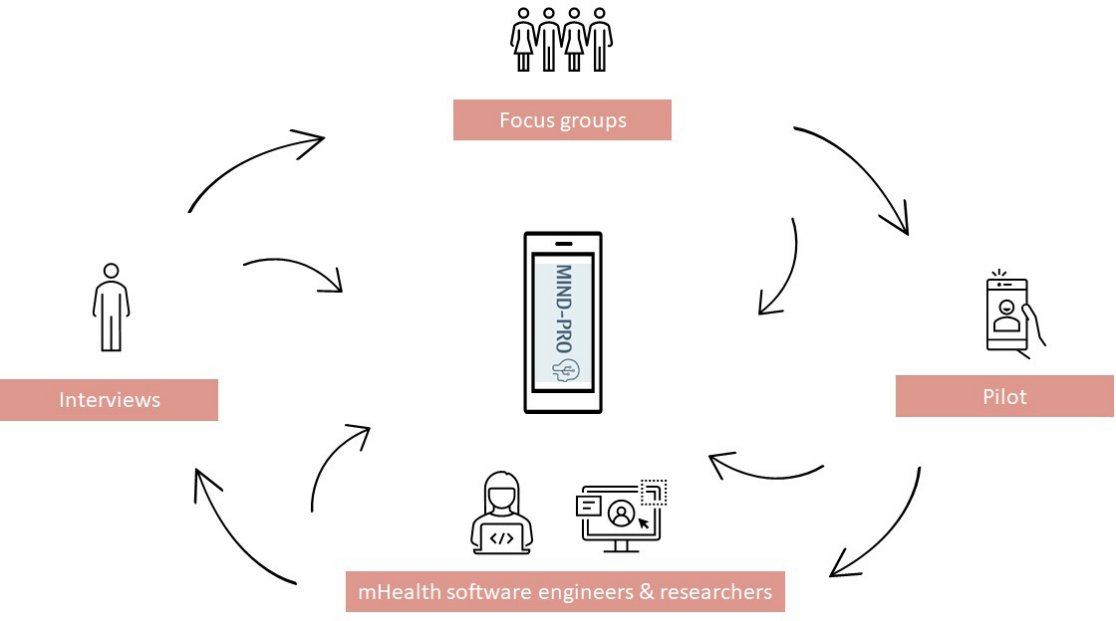
Design process of the intervention. In this figure, the process of co-creating the intervention is illustrated. The researchers, mHealth software engineers and end-users worked in iterative cycles of feedback to co-create the app. Semi-structured interviews, focus groups and a pilot phase were conducted to identify barriers and facilitators, which led to adaptations to the app. mHealth, Mobile Health; MIND-PRO, mHealth Intervention for Dementia Prevention through lifestyle Optimisation.

During the development process of the app, our target population and mHealth software engineers were actively involved, as shown in figure 1. The initial concept of the MIND-PRO app was based on insights from prior eHealth and mHealth trials conducted by our research team, specifically the from HATICE^16^ and PRODEMOS^20^ studies. The app was subsequently refined through iterative feedback cycles, resulting in an optimised version tailored to the needs and wishes of the target population for use in this trial. To achieve this, we conducted semi-structured interviews and focus groups to gain a deeper understanding of participants’ perspectives and their requirements for a coach-supported lifestyle intervention.

### Primary and secondary outcomes

Following a type 2 hybrid effectiveness-implementation design, equal weight will be placed on the effectiveness and implementation outcomes. The primary effectiveness outcome will be the difference between baseline and 12 months follow-up of a composite z-score of systolic blood pressure, non-high-density lipoprotein (HDL) cholesterol and body mass index (BMI). The secondary effectiveness outcomes include changes from baseline in CAIDE dementia risk score,^21^ individual modifiable components of the CAIDE risk score, questionnaires on physical activity,^22^ disability,^23^ depressive symptoms^24^ and cognitive functioning,^25 26^ cost-effectiveness analysis including intervention costs and daily distance moved measured by Behapp. Depressive symptoms are an outcome, because indirect effects may occur, for example, through increased physical activity. Cognitive functioning will be tested with The Rowland Universal Dementia Assessment Scale (RUDAS) and the Box Task for a computerised assessment of visuospatial working memory.^27^ Two parallel forms of the RUDAS were created for repeated administration at the beginning and end of the trial. Daily distance moved will be measured using a separate smartphone application (Behapp), which is optional and for which separate informed consent will be asked. Behapp has been developed and is operated by The University of Groningen to provide a remote, unobtrusive and objective measure of overall social mobility in a longitudinal daily-life manner. An in-depth explanation of Behapp, as well as the privacy and security measures, deployed by Behapp are described in detail previously.^28^ Effectiveness outcomes are summarised in table 1.

**Table 1:** Effectiveness outcomes

Primary outcomes
Composite score of systolic blood pressure, non-HDL cholesterol and body mass index (BMI)

Secondary outcomes	Measurement tool
(1) Physical exercise	Self-administered short International Physical Activity Questionnaire-Short Form^22^
(2) Disability	WHO Disability Assessment Schedule 2.0 (12-item)^23^
(3) Depressive symptoms	Geriatric Depression Scale 15-item^24^
(4) CAIDE dementia risk score	Cardiovascular Risk Factors, Ageing and Dementia^21^
(5) Individual modifiable components of the CAIDE risk score	Age (≥47 years), education (<10 years), sex, systolic blood pressure, obesity (BMI ≥30), physical activity and total cholesterol
(6) Intervention costs	Costs of coaches and app intervention development
(7) Cost-effectiveness	Cost-effectiveness analyses are clarified in online supplemental appendix 1
(8) Cognitive functioning	The Rowland Universal Dementia Assessment Scale^26^ and the Box Task^27^
(9) Daily distance moved	Behapp, a remote behavioural monitoring app^28^

The implementation of the intervention will be measured using implementation outcomes reflecting coverage, acceptability, adoption, appropriateness, feasibility, fidelity, costs and sustainability.^29^ All implementation outcomes and evaluation methods are shown in table 2.

**Table 2:** Summary of implementation research methods and outcomes

Method	Implementation outcome^7^	Measurements	Population	When measured
Quantitative	Coverage	(Non)response rates, comparison characteristics participants with eligible/source population	Potential target population	At baseline
	Adoption	Quantitative analysis of the utilisation, usage and uptake—for example, logins, goals setting, sending messages	All intervention participants	After 1 month (data features)
	Appropriateness	Short questionnaire of perceived fit or relevance*	All intervention participants	After 6 months and at study end
	Acceptability	Short questionnaire of agreeability, user-friendliness, credibility, complexity, content*	All Intervention participants	After 6 months and at study end
	Feasibility	The extent to which the mHealth intervention can be carried out	All intervention participants	After 6 months and at study end
	Sustainability	Adherence, dropout, data features (time spent in the app)	All intervention participants, dropouts	Data features throughout the study
	Implementation cost	Implementation costs (see cost-effectiveness analysis plan in online supplemental appendix 1)	n.a.	After 6 months and at end of study
Qualitative	Appropriateness	Perceived fit or relevance of the intervention	Intervention participants†; coaches	After 6 months
	Adoption	Initial utilisation, usage and uptake	Intervention participants†	After 6 months
	Acceptability	Agreeability, user-friendliness, credibility, complexity, content	Intervention participants†; coaches	After 6 months
	Feasibility	Practical and social barriers/facilitators related to the feasibility of the intervention	Intervention participants†; coaches	After 6 months
	Fidelity	Degree to which the mHealth application is implemented compared with the original protocol	Coaches	After 6 months
	Sustainability	Adherence	Coaches	After 6 months

* We used several questions from the validated mHealth App Usability Questionnaire for Interactive mHealth Aapps (MAUQ) (), where applicable to our intervention.^38^

† 30 participants (10 Dutch, 10 Turkish, 10 South-Asian Surinamese) will be invited for an interview on the implementation outcomes.

mHealthMobile Healthn.a.not available

### Study procedures

**Figure 2:**
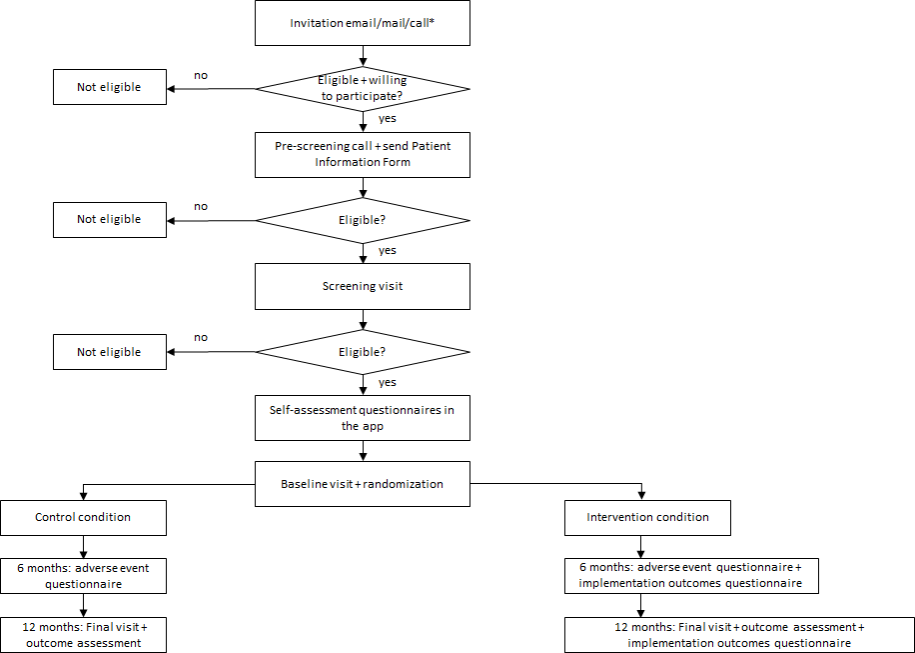
Flow chart of study procedure. *An initial invitation will be sent to potentially eligible participants via mail. If there is no response, a second invitation will be sent via mail or email. If there is still no response, we will call the participants to ask if they are willing to participate.

The study procedures are visualised in figure 2. Potentially eligible participants, based on information from the HELIUS study, will be invited via a letter in Dutch and Turkish to participate in the MIND-PRO study starting from July 2024 and ending in July 2026. The planned recruitment period is 12 months. We expect to be finished with recruitment in July 2025. A second invitation mail or email will be sent to those who did not respond to the first invitation. When there is still no response, we will call the potentially eligible participants to ask whether they want to participate. Those who are willing to participate will undergo a prescreening call to assess their eligibility for a further screening visit and subsequent baseline visit. During the screening visit, written informed consent will be obtained and additional measurements (weight, blood pressure, non-HDL cholesterol and assessment of cognitive functioning) will be conducted. Also, data on medication (antihypertensives, cholesterol-lowering drugs, diabetes treatments and antidepressants) will be collected. At the end of the screening visit, the MIND-PRO app will be downloaded with an explanation how to fill in the self-assessment questionnaires in the app. Participants will be asked to fill out the questionnaires themselves in the app. After the screening visit, the baseline visit will take place during which the self-assessment questionnaires will be checked and completed and the participant will be randomised to the intervention or control group, stratified by ethnicity. Six months after randomisation, participants will be requested to fill out a questionnaire on adverse events, which will be sent via the app. They will also be asked about their healthcare utilisation, specifically general practitioner visits, dietitian or physiotherapist visits and hospital visits. Additionally, all participants in the intervention group will be asked to fill out a questionnaire on implementation outcomes in the app 6 months after randomisation. Those who do not fill out the questionnaires will be called and the questionnaires will be administered by an assessor by telephone. For a more in-depth, qualitative assessment of implementation outcomes, a purposive sample from the intervention group of approximately 30 participants (10 from each ethnic group) will be asked to participate in an interview (table 2). At the end of the study, the questionnaires, medication use, healthcare utilisation and measurements performed during the screening visit will be repeated. Assessors will be matched to the participants’ ethnicity as much as possible.

### Randomisation and blinding

Randomisation will take place in the app using a computer algorithm in a 1:1 manner, stratified by ethnicity. Randomisation will be carried out by trained assessors at the baseline visit. Participants will be informed that they are randomised to one of two different smartphone apps that can provide support in changing their lifestyle to reduce the risk of dementia. Partners or first-degree family members who also participate in the MIND-PRO study will automatically be allocated to the same treatment arm to limit contamination. Outcome assessment will be performed by an assessor who is blinded to treatment allocation. The coaches who support participants in behaviour change are, due to the nature of the intervention, not blinded. A certain level of unblinding during outcome assessment is conceivable, since a participant could share details about participation in the MIND-PRO app specifically to the outcome assessor. Participants and assessors are therefore instructed to not discuss the intervention with each other. If any unblinding occurs, its impact is likely limited since our primary outcome consists solely of objectively measured parameters.

### Safety considerations

Use of the mHealth intervention is not expected to be associated with health risks beyond those typically experienced by individuals leading an active lifestyle. Considering the low risk, no Data Safety Monitoring Board or Safety Committee will be installed. Multiple measures will be taken to reduce any potential safety risk. First, it will be explicitly communicated that the MIND-PRO app does not replace any regular primary or secondary care. Second, if any condition requiring medical attention arises during the study (eg, hypertension that qualifies for treatment according to clinical guidelines, as observed during the measurements at the screening visit), participants will be referred to their own doctor by the assessor, coach or the study investigator. Third, adverse events will be checked using a questionnaire at 6 months, for which participants will receive notifications on their smartphone and reminders through email. Fourth, the MIND-PRO app is built in accordance with the highest security requirements in healthcare and complies with NEN 7510, the Health Insurance Portability and Accountability Act and General Data Protection Regulation (GDPR).

## Statistical analysis and quality assurance

### Sample size

The sample size was calculated based on the expected effect of the intervention on our primary effectiveness outcome in individuals with low education, used as a proxy for low SES, from our previous prevention trials. In participants with a low educational level in the HATICE trial,^16^ the mean difference between intervention and control participants in change from baseline was 0.107 (pooled SD 0.454) for the composite z-score after 1.5 years. In the preDIVA trial,^30^ the difference in composite z-score between individuals with a low educational level who developed dementia and those who did not during 6–8 years of intervention was 0.096 after 24 months. Therefore, a mean difference in change of 0.107 was considered as potentially clinically relevant with respect to the risk of dementia. For the effect of the intervention within specific migrant groups, no data are available. With 277 participants per treatment arm, we will have 80% power (with alpha set at 0.05) to detect a 0.107 (pooled SD 0.454) difference on our primary effectiveness outcome. To adjust for an anticipated drop-out of 20% in this specific target population, 692 participants will be recruited.

### Data analysis

The primary effectiveness outcome is the difference between the composite z-score after 12 months and the baseline composite z-score ((z_SBP_+z_non-HDL cholesterol_+z_BMI_)/3). The baseline mean and SD will be used to calculate composite z-scores at both time points to be able to detect a change. For the primary analyses, we will use a linear mixed effects model with individual observations nested within ethnic groups, operationalised by a random intercept and/or slope (depending on best model fit). If needed, we will explore the impact of any baseline imbalances by including imbalanced factors as a covariate in a sensitivity analysis. A per-protocol analysis will be done, based on uptake within the first month and continued use during the whole intervention period. Exploratory subgroup analyses on the primary outcome will be performed for ethnic group, sex, age group, history of CVD and diabetes.

We will conduct a sensitivity analysis using multiple imputation for the primary effectiveness outcome. Implementation of the intervention will be analysed with a series of implementation outcomes, as summarised in table 2. We will investigate frequency of app use and contact with the coach in relation to the effect on the primary outcome. We will graphically portray the Likert scales for the questionnaires and perform thematic analysis for the in-depth interviews. The interview guides can be found in the online supplemental appendix 3.

For the secondary outcomes, the effect on the CAIDE dementia risk score and its individual components (ie, blood pressure, BMI, total cholesterol, physical activity) will be analysed using the same model as for the primary analysis. In addition, as an exploratory analysis, the effect on daily changes in general movement will be assessed based on the data collected with the BeHapp application.^28 31 32^ For that purpose, time series analysis will be performed to detect trends in this longitudinal data as a function of intervention, accounting for clustering within ethnicities.

Scales for disability, depressive symptoms and cognitive functioning, mostly ordinal, will also be analysed as linear scales if the data characteristics allow. The same model as used for the primary analysis will be employed. Poisson regression or zero-inflated models may be applied to distributions resembling count or zero-inflated data. A description of the cost-effectiveness analysis can be found in online supplemental appendix 1.

### Handling and storage of data

The study will be conducted according to the principles of the Declaration of Helsinki (version of 2013) and in accordance with the Dutch Medical Research Involving Human Subjects Act and the GDPR

Data management will take place centrally at the Department of Public and Occupational Health (Amsterdam UMC, location AMC) in close collaboration with the app developer, and personal data will be archived and stored 15 years after the end of the study, in accordance with Good Clinical Practice guidelines. Hosting of the mHealth platform, which also includes data management for all data collected in the project (the case report form is integrated in the platform), will take place at a secure server in the Netherlands, hosted by a certified Microsoft datacenter which is compatible with the highest standards of data management in medical research. For data cleaning and analysis, only pseudonymised data will be stored at the secure server in the Netherlands, in line with the current GDPR regulations. Participant’s data are coded with a unique number (participant identification number). The key to this code is known by the coordinating research centre. The coordinator at Amsterdam UMC and one senior researcher will have access to this code. Monitoring of the study will be done by the Clinical Monitoring Center of the Amsterdam UMC.

## Discussion

The rationale for our target group, individuals aged 50–75 years of low SES and/or migration background with elevated dementia risk factors or CVD, is twofold. First, most dementia risk factors exert their strongest detrimental effect in midlife, therefore optimising these risk factors in (late) midlife, rather than late-life is likely to be more effective for the prevention of cognitive decline and old-age dementia.^33 34^ Second, as the prevalence of dementia in high-income countries is higher among people of low SES and/or migration background, there is more room for reducing dementia risk factors in these populations.^35 36^

Recruiting participants from the HELIUS cohort has its advantages, such as feasibility, but it also introduces potential selection bias. Although demographic differences between the baseline population and the general population were limited,^19^ these differences may have widened over time and participants may be healthier and more motivated to change their lifestyles, a common challenge in preventive studies that complicates generalisability. Additionally, by narrowing our focus to three specific ethnic groups, we acknowledge that the findings of this study may not be applicable to other ethnic populations. However, this approach was chosen for practical feasibility, enabling us to focus on groups with the highest risk of dementia, based on measured risk factors within the HELIUS study.

Furthermore, in the proposed study design, we chose ethnically matched lifestyle coaches because they are likely more attuned to the preferences and needs within their cultural context. This approach mirrors common practices in Dutch primary care, where GPs often employ ethnically matched nurses to enhance the acceptability and perceived appropriateness of lifestyle guidance in cardiovascular risk management.^37^

Focusing on risk reduction is important, as even a modest reduction in dementia risk factors at the individual level could translate into a substantial decrease in dementia incidence at the population level.^5^ A crucial inquiry in risk reduction is how to effectively devise and execute strategies that promote a healthy lifestyle, thereby mitigating dementia risk, particularly among the most vulnerable groups, and preventing the exacerbation of health inequalities. This study aims to offer tangible evidence concerning the potential impact on these under-represented high-risk individuals, while also uncovering key implementation strategies and challenges relevant to diverse populations.

Given the nature of the intervention and the close-knit communities in which our target groups reside, full blinding not feasible, which may lead to contamination. It is important to take this into account, but hard to predict how, and if, contamination between study arms will affect the results of the trial.

Apart from that, we will disclose the measurements conducted during the screening visit to all participants. This may trigger heightened awareness and motivation in both study arms. For example, the identification of hypertension will prompt treatment initiation in both the control and intervention groups. However, a similar procedure of disclosing the measurement results was also employed in the HATICE^16^ and the preDIVA^30^ study, which showed that this effect is small. By using both these studies to calculate our sample size, a potential overestimation of the expected contrast between study groups is mitigated.

Finally, we do not measure dementia incidence as an outcome, even though the trial focuses on reducing dementia risk factors. This would require a much longer follow-up period and larger sample size. This precludes the establishment of a potential causal link between this lifestyle intervention and the development of dementia. Even for a clinically relevant change in cognitive function, a much larger and longer study would be needed. However, we do assess cognition in our study, to evaluate the applicability and feasibility of longitudinal assessment using a test battery specifically designed for those with low literacy.

In conclusion, the findings of our proof-of-concept trial, which examines both effectiveness and implementation, have the potential to inform the future development of culture-sensitive lifestyle interventions and large-scale RCTs tailored to address dementia risk reduction strategies for individuals with low SES and/or a migration background.

## supplementary material

10.1136/bmjopen-2024-088324 online supplemental file 1

## References

[1] Nichols E, Steinmetz JD, Vollset SE (2022). Estimation of the global prevalence of dementia in 2019 and forecasted prevalence in 2050: an analysis for the Global Burden of Disease Study 2019. Lancet Public Health.

[2] Livingston G, Huntley J, Sommerlad A (2020). Dementia prevention, intervention, and care: 2020 report of the Lancet Commission. Lancet.

[3] Zlokovic BV, Gottesman RF, Bernstein KE (2020). Vascular contributions to cognitive impairment and dementia (VCID): A report from the 2018 National Heart, Lung, and Blood Institute and National Institute of Neurological Disorders and Stroke Workshop. Alzheimers Dement.

[4] Selten J-P, Termorshuizen F, van Sonsbeek M (2021). Migration and dementia: a meta-analysis of epidemiological studies in Europe. Psychol Med.

[5] Barnes DE, Yaffe K (2011). The projected effect of risk factor reduction on Alzheimer’s disease prevalence. Lancet Neurol.

[6] Shiekh SI, Cadogan SL, Lin LY (2021). Ethnic Differences in Dementia Risk: A Systematic Review and Meta-Analysis. J Alzheimers Dis.

[7] Steyaert J, Deckers K, Smits C (2021). Putting primary prevention of dementia on everybody’s agenda. *Aging & Mental Health*.

[8] Stowell E, Lyson MC, Saksono H (2018). Designing and evaluating mhealth interventions for vulnerable populations.

[9] Latulippe K, Hamel C, Giroux D (2017). Social Health Inequalities and eHealth: A Literature Review With Qualitative Synthesis of Theoretical and Empirical Studies. J Med Internet Res.

[10] Bol N, Helberger N, Weert JCM (2018). Differences in mobile health app use: A source of new digital inequalities?. *The Information Society*.

[11] Watchman K (2020). Overcoming ethical challenges affecting the involvement of people with dementia in research: recognising diversity and promoting inclusive research.

[12] Eggink E, Hafdi M, Hoevenaar-Blom MP (2022). Attitudes and views on healthy lifestyle interventions for the prevention of dementia and cardiovascular disease among older people with low socioeconomic status: a qualitative study in the Netherlands. BMJ Open.

[13] Zhang J, Eggink E, Zhang X (2022). Needs and views on healthy lifestyles for the prevention of dementia and the potential role for mobile health (mHealth) interventions in China: a qualitative study. BMJ Open.

[14] Hafdi M, Eggink E, Hoevenaar-Blom MP (2021). Design and Development of a Mobile Health (mHealth) Platform for Dementia Prevention in the Prevention of Dementia by Mobile Phone Applications (PRODEMOS) Project. Front Neurol.

[15] van Middelaar T, Beishuizen CRL, Guillemont J (2018). Engaging older people in an internet platform for cardiovascular risk self-management: a qualitative study among Dutch HATICE participants. BMJ Open.

[16] Richard E, Moll van Charante EP, Hoevenaar-Blom MP (2019). Healthy ageing through internet counselling in the elderly (HATICE): a multinational, randomised controlled trial. Lancet Digit Health.

[17] Barbera M, Mangialasche F, Jongstra S (2018). Designing an Internet-Based Multidomain Intervention for the Prevention of Cardiovascular Disease and Cognitive Impairment in Older Adults: The HATICE Trial. *J Alzheimers Dis*.

[18] Landes SJ, McBain SA, Curran GM (2020). Reprint of: An introduction to effectiveness-implementation hybrid designs. Psychiatry Res.

[19] Snijder MB, Galenkamp H, Prins M (2017). Cohort profile: the Healthy Life in an Urban Setting (HELIUS) study in Amsterdam, The Netherlands. BMJ Open.

[20] Moll van Charante EP, Hoevenaar-Blom MP, Song M (2024). Prevention of dementia using mobile phone applications (PRODEMOS): a multinational, randomised, controlled effectiveness-implementation trial. Lancet Healthy Longev.

[21] Kivipelto M, Ngandu T, Laatikainen T (2006). Risk score for the prediction of dementia risk in 20 years among middle aged people: a longitudinal, population-based study. Lancet Neurol.

[22] Committee IR (2005). Guidelines for data processing and analysis of the International Physical Activity Questionnaire (IPAQ)-short and long forms. http://wwwipaqkise/scoringpdf.

[23] Üstün TB (2010). Measuring health and disability: Manual for WHO disability assessment schedule WHODAS 2.0.

[24] Sheikh JI, Yesavage JA (2014). Geriatric depression scale (GDS): recent evidence and development of a shorter version.

[25] Goudsmit M, van Campen J, Schilt T (2018). One Size Does Not Fit All: Comparative Diagnostic Accuracy of the Rowland Universal Dementia Assessment Scale and the Mini Mental State Examination in a Memory Clinic Population with Very Low Education. Dement Geriatr Cogn Dis Extra.

[26] Storey JE, Rowland JTJ, Basic D (2004). The Rowland Universal Dementia Assessment Scale (RUDAS): a multicultural cognitive assessment scale. Int Psychogeriatr.

[27] Kessels RPC, Postma A (2018). The Box Task: A tool to design experiments for assessing visuospatial working memory. Behav Res Methods.

[28] Jagesar RR, Vorstman JA, Kas MJ (2021). Requirements and Operational Guidelines for Secure and Sustainable Digital Phenotyping: Design and Development Study. J Med Internet Res.

[29] Peters DH, Adam T, Alonge O (2013). Implementation research: what it is and how to do it. BMJ.

[30] van Charante EPM, Richard E, Eurelings LS (2016). Effectiveness of a 6-year multidomain vascular care intervention to prevent dementia (preDIVA): a cluster-randomised controlled trial. Lancet.

[31] Jongs N, Jagesar R, van Haren NEM (2020). A framework for assessing neuropsychiatric phenotypes by using smartphone-based location data. Transl Psychiatry.

[32] Eskes P, Spruit M, Brinkkemper S (2016). The sociability score: App-based social profiling from a healthcare perspective. Comput Human Behav.

[33] van Dalen JW, Brayne C, Crane PK (2022). Association of Systolic Blood Pressure With Dementia Risk and the Role of Age, U-Shaped Associations, and Mortality. JAMA Intern Med.

[34] Martín-Ponce E, Santolaria F, Alemán-Valls MR (2010). Factors involved in the paradox of reverse epidemiology. Clin Nutr.

[35] Parlevliet JL, Uysal-Bozkir Ö, Goudsmit M (2016). Prevalence of mild cognitive impairment and dementia in older non-western immigrants in the Netherlands: a cross-sectional study. Int J Geriatr Psychiatry.

[36] Bodryzlova Y, Kim A, Michaud X (2023). Social class and the risk of dementia: A systematic review and meta-analysis of the prospective longitudinal studies. Scand J Public Health.

[37] van Apeldoorn JAN, Roozekrans AK, Harskamp RE (2024). General practitioners’ views on cardiovascular prevention for ethnic minorities—a qualitative study in the Netherlands. Fam Pract.

[38] Zhou L, Bao J, Setiawan IMA (2019). The mHealth App Usability Questionnaire (MAUQ): Development and Validation Study. JMIR Mhealth Uhealth.

