# Supplementary material for: mHealth Intervention for Dementia Prevention through lifestyle Optimisation (MIND-PRO) in a primary care setting: protocol for a randomised controlled trial in people with low SES and/or migration background
Source: BMJ Open. 2025 Feb 3;15(2):e088324. doi: 10.1136/bmjopen-2024-088324 (PMC11956295; doi:10.1136/bmjopen-2024-088324)
Supplement: online supplemental file 1 [file bmjopen-15-2-s001.docx]

**Appendix 1 – Health Economics Analysis**

A key challenge in dementia primary prevention interventions in midlife or early late-life is the long duration before an effect is expected on the outcome dementia. Mathematical model-based simulation studies can be used to extrapolate short-term trial outcomes by synthesizing data from multiple sources (e.g., trial for effectiveness on risk factor and cohort studies on association between risk factor and dementia onset). The health economic evaluation in this study is closely linked to a project of the parallel BIRD-NL consortium. In BIRD-NL, a health economic microsimulation model will be developed based on the existing models MISCAN (dementia) and PRODEMOS (dementia and cardiovascular disease) studies. The basis of this model is a dementia-free survival function and a mortality function, both by age, sex and SES. Updated evidence on the association between modifiable risk factors and dementia onset, and non-modifiable factors (migration background) and dementia onset will be used to adjust the dementia-free survival function. Possible interactions with age, sex and SES will be included in the model. The model will simulate the annual incidence of dementia onset and death, and produce the outcomes life expectancy, person-years with dementia, QALYs and costs.

The mHealth intervention will be implemented into the model (after basic model development in the BIRD-NL consortium). This will be done in terms of the target population (Dutch persons aged 50+ with low SES and/or migration background) and intervention effect. The model will simulate the dementia onset in the usual care strategy of individuals using the relative risk corresponding to each factor’s risk status. The mHealth intervention effect is reflected by copying the baseline population characteristics and apply the effect observed from the current trial on each modifiable risk factor (e.g., a difference in score between baseline and follow-up, between control and intervention arm). The model will simulate the 'mHealth in addition to usual care’ strategy using the same procedure as for the usual care strategy.

As the current RCT only covers a 1-year follow-up period, experts within the NDPI consortium will be asked for evidence (or, if unavailable, their opinion) on plausible estimates of long-term effectiveness endurance beyond the trial follow-up period. These assumptions will be implemented in the model. Intervention costs will be obtained from estimates on coaching time and mHealth app operationalization costs recorded as secondary outcome during this trial.

These simulations will result in the proportion of the starting population with a history of dementia and mortality (and cardiovascular disease) over lifetime. The simulated data will be combined with estimates from literature on health-related quality of life and care costs related to dementia (and cardiovascular disease) to estimate the cumulative lifetime QALYs and costs for the 'usual care’ strategy and ‘mHealth in addition to usual care’ strategy. This allows to estimate the incremental cost-effectiveness ratio of the mHealth intervention.

Uncertainty will be addressed by univariate sensitivity analysis (e.g., using different assumptions on beyond-trial effectiveness endurance).

Results will be published open-access following international CHEERSII reporting guidelines.

**Appendix 2 – Informed Consent Form**

**Disclaimer: This is a not a certified translated version of the original Dutch Patient Information form and Informed Consent form. The Medical Ethics Committee of Amsterdam UMC only gave permission to use the Dutch version of this form.**

**Information for participation in medical research**

**MIND-PRO study: Healthier brains through a healthier lifestyle – research with an app.**


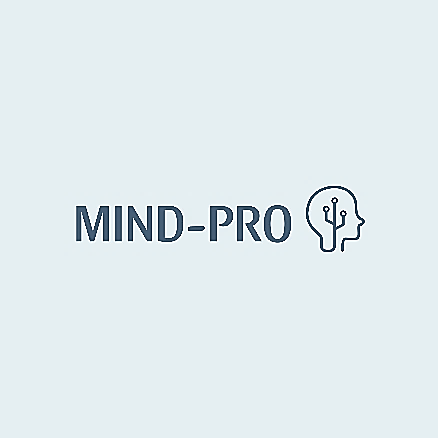


**Introduction**

Dear Sir/Madam,

We would like to ask you whether you would be interested in participating in medical research. Participation is voluntary.
In this information letter you can read what kind of research is involved, what it means for you, and what the advantages and disadvantages are. It's a lot of information. You can read through the information and decide if you want to participate. If you would like to participate, please complete the form in Appendix B together with the investigator.

**Ask your questions**

You can make your decision with the information you will find in this information letter. In addition, we recommend that you do this:

- Ask questions to the researcher who gives you this information.

- Talk to your partner, family, or friends about this research.

- Ask questions to the independent expert. For contact details see Appendix A

- Read the information on [www.rijksoverheid.nl/mensenonderzoek](http://www.rijksoverheid.nl/mensenonderzoek).

1. **General information**

The Amsterdam UMC coordinates this study. In the following, we always refer to the Amsterdam UMC as the 'sponsor’. Researchers, which may also include doctors, coaches, and research nurses, conduct the study. The government pays for this research.

Participants in a medical-scientific study are often referred to as research subjects. Both patients and people who are healthy can be research subjects.

This study requires 700 subjects between the ages of 50 and 75.

The medical ethics review committee of Amsterdam UMC has approved this study.

**2. What is the purpose of the study?**

The aim of this research is to keep the brain healthy by living a healthier live. In this study, we will investigate whether the use of a special app can help with this.

1. **What is the background to the research?**

Did you know that you can keep your brain healthy for longer by changing your lifestyle? Examples of an unhealthy lifestyle are smoking, obesity, lack of exercise or an unhealthy diet. By working on these things, you can not only improve your heart health, but probably brain health as well. For many people, it is not easy to start living a healthier life. That's why we've created an app to help you with this. In this study, we want to test whether this app also works in daily life. The app and the guidance by a coach during the study have been specifically developed for people of Turkish, Hindustani and Dutch origin.

1. **How does the research work?**

*How long does the study take?*

The study will last a total of 12 months.

*Step 1: Are you suitable to participate?*

We first want to know if you are suitable to join the study. Therefore, the researcher will first ask several questions by telephone about:

- Your health and whether you have or have had any illnesses.
- Medication use
- Owning a smartphone
- Understanding of the Dutch language

If the investigator thinks you are likely to be suitable to participate, we will invite you for a visit. This visit will take place at a research centre near you, at the Amsterdam UMC or at your home. The visit lasts approximately 1 hour. We then carry out the following examinations:

- Measuring Blood Pressure
- Measuring weight
- Measuring cholesterol (using a finger prick)
- You install the app on your own phone
- You fill out questionnaires at home. The questionnaires include physical activity, mood and self-efficacy

*Step 2: Using the app*You will be using the app for 12 months.

We created two distinct version of the app for this research; the version you receive is decided by a lottery.

*Step 3: Surveys and measurements*

After this, we invite you for a second visit. This visit will take place at a research centre near you, at the Amsterdam UMC or at your home. The visit lasts about 30 minutes.

This is what we will do during this visit:

- We check whether all questionnaires have been completed
- Via a drawing, we will determine which version of the app you will use.
- Explanation on how to use the app.

Six months after the start of the study, we will send you a questionnaire. You can fill in the questionnaire via the app. We ask if you have been to the doctor or if you have fallen ill. It will take you about 10 minutes to complete this questionnaire.

At the end of the study, we will invite you for the last time for a visit of about 30 minutes at a research center near you, at the Amsterdam UMC or at your home.

We then take the following measurements:

- We will measure your weight and blood pressure again.
- We measure your cholesterol again with a finger prick.

You will be asked to fill out the same questionnaires as at the beginning of the study.

*What is different from regular care?*

Participating in the study has no consequences for the regular care you receive from, for example, your GP. The use of the app and the checks that are part of this study are extra.

1. **What agreements do we make with you?**

We want the research to go well. That is why we make the following agreements with you:

- You use the app in the way the researcher explained.
- You come to every appointment, or contact us if it doesn't work out.
- You contact the investigator in these situations:
  - You no longer want to participate in the study.
  - Your phone number, address or e-mail address changes

1. **Adverse effects**

*What are the disadvantages of participating in the study?*

The disadvantage of participating in this study is the extra time it takes.

The finger prick may hurt for a while.

1. **What are the advantages and disadvantages of participating in the study?**

Participating in the study can have advantages and disadvantages. We list them below. Think about this carefully, and talk about it with others.

Using the app could potentially help you live a healthier life, but we don't know for sure. With your participation, you help the researchers gain more insight into how the app works and its influence on a healthier lifestyle.

Participating in the study can have these disadvantages or consequences:

- - You may be bothered by the measurements during the examination (tight blood pressure band, finger prick).
  - Participating in the study will cost you extra time.
  - You are requested to adhere to the agreements that are part of the study.
  - The questionnaires can sometimes be difficult, for example when it comes to questions about your mood

*Don't want to participate?*

You decide whether you want to participate in the study. Don't want to participate? Then there will be no change in the usual care you are currently receiving.

1. **When does the study end?**

The investigator will let you know if there is new information about the study that is important to you. The researcher will then ask you whether you would want to continue to participate.

In the following situations, the investigation will stop for you:

- All the investigations according to the schedule are over.
- You want to stop the research yourself. You can do so at any time. Then report this to the researcher immediately. You don't have to tell them why you're quitting.
- The researcher thinks it is better for you to stop. The researcher will then explain why.
- One of the following authorities decides that the investigation must stop:
  - Amsterdam UMC
  - the government, or
  - the medical ethics committee that assesses the research.

*What happens if you stop the study?*

The investigators will use the data collected up to the time of discontinuation.

The entire study is over when all participants are finished.

1. **What happens after the study?**

You will not be able to continue using the app you used during the study after the study.

*Will you get the results of the study?*

About a year after the study is completed, the investigator will be able to let you know what the main outcomes are. If you wish, you can indicate this in the consent form. The researcher will also be able to tell you which version of the app you used. Don't want to know? Then tell the researcher. He/she won't tell you.

1. **What do we do with your data?**

Will you participate in the study? Then you also consent to the collection, use and storage of your data.

*What data do we store?*

We store this data:

- your contact details: address, email address, telephone number, contact details GP

- your identity data: name, date of birth, gender, ethnicity

- data about your health

- (medical) data we collect during the study

*Why do we collect, use and store your data?*

We collect, use and store your data in order to be able to answer the questions of this study. And to be able to publish the results.

*How do we protect your privacy?*

To protect your privacy, we give your data a code instead of your identifiable data. We will put this code on all your data. We store the key to the code in a secure place at the Amsterdam UMC. When we process your data, we only use that code at all times. Also in reports and publications about the research, no one can recall that it was about you.

*Who can see your data?*

However, some people will be able to see your name and other personal information without a code. This may include data collected specifically for this study.

These are people who check whether the researchers are conducting the research properly and reliably. These people will be able to view your data:

- Members of the committee that monitors the security of the investigation.
- An inspector hired by the sponsor.
- National supervisory authorities, such as the Health Care Inspectorate.

These people will keep your information confidential. We ask for your permission for access by these persons.The Health and Youth Care Inspectorate can view your data without your permission.

*How long do we keep your data and bodily material?*

We store your data for up to 15 years after the end of the study at Amsterdam UMC.

Your blood (finger prick) will be destroyed immediately after use.

*Can we use your data for other research?*

Your data collected within this study may also be important for other scientific research on lifestyle and brain health. Therefore, your data will be stored at Amsterdam UMC for 15 years. In the consent form, you indicate whether you are okay with this. What happens if you don’t give permission? Then you can still participate in this study. You will receive the same care.

*What happens in the event of unexpected discoveries?*

During the examination, we may find something that is not directly important for the examination but is important for your health, such as high blood pressure. The investigator will advise you to contact your general practitioner or specialist. The costs of this are covered by your own health insurance. A visit to the GP is not covered by the deductible. With the form, you give permission for your GP or specialist to be informed.

*Can you revoke your consent to the use of your data?*

You can revoke your consent to the use of your data at any time. Then tell the researcher. If you revoke your consent, your data and the data collected will also be deleted.

*Would you like to know more about your privacy?*

- Would you like to know more about your rights in the processing of personal data? Then take a look at [www.autoriteitpersoonsgegevens.nl](http://www.autoriteitpersoonsgegevens.nl).
- Do you have questions about your rights? Or do you have a complaint about the processing of your personal data? Please contact the person responsible for processing your personal data. For your research, that is:
  - Amsterdam UMC. See the end of this docuement for contact details, and website.
- If you have any complaints about the processing of your personal data, we recommend that you first discuss them with the investigation team. You can also go to the Data Protection Officer of the Amsterdam UMC. Or you can file a complaint with the Dutch Data Protection Authority.

*Where can you find more information about the study?*

If you would like more information about the study, please contact the investigators. You can find the contact details at the bottom of this form.

1. **Will you be compensated if you participate in the study?**

Participation in the study is free of charge. You will also not be compensated if you participate in this study. However, you can get reimbursement for your (extra) travel expenses in the form of a GVB day ticket or via a claim form if you come by car. You will receive this for each time you have to travel to the study site or the Amsterdam UMC for a visit for this study.

1. **Are you insured during the examination?**

You are not insured for this examination. Because participating in the study has no additional risks. Therefore, the commissioner of the reviewing committee does not have to take out additional insurance.

1. **We inform your GP**

The investigator will send your GP a letter/email to let them know that you are participating in the study. This is for your own safety. We may also contact your doctor or general practitioner, for example about your medical history or about the medicines you are taking.

1. **Do you have any questions?**

If you have any questions about the study, please contact the research team. Do you want advice from someone who has no interest in it? Then go to the independent expert, for contact details see Appendix A. He knows a lot about the investigation, but does not cooperate with this investigation.

Do you have a complaint? Discuss this with the researcher or doctor who is treating you. Would you rather not do this? Then go to the hospital's complaints committee. Appendix A tells you where to find them.

1. **How do you give permission for the study?**

You can first think about this research calmly. During the first visit, you will tell the investigator if you understand the information and if you will participate. After that, you will fill out the consent form. An example of the consent form is attached to this information letter. You and the investigator will both receive a signed version of the consent form.

Thank you for your time.

**Contact details for Amsterdam UMC**

Researcher:

Eric Moll van Charante, Principal Investigator

Research Coordinator:

Anne Roos van der Endt

Independent expert:

Ralf Harskamp, general practitioner

Complaints:

If you have any questions or complaints, we recommend that you contact the investigators first.

If this is not possible for you, you can make use of the services of the complaints officer of the Patient Information and Complaints Reception Department. The Patient Information & Complaints Reception department is located on the ground floor of the outpatient clinic (A0). It is open on weekdays from 9:00 a.m. to 12:30 p.m. and 1:00 p.m. to 3:30 p.m. Then the department can also be reached by phone (020 566 3355). If the department is closed, you can leave a voicemail message or send an e-mail. The department's email address has been [patientenvoorlichting@amc.nl](mailto:patientenvoorlichting@amc.nl).

Data Protection Officer of the institution:
If you have any questions or comments about the protection of your privacy, you can contact the Data Protection Officers of Amsterdam UMC via privacy@amsterdamumc.nl

**Subject Consent Form**

Belonging to

**MIND-PRO study: Healthier brains through a healthier lifestyle – research with an app.**

- I have read the information letter. I was also able to ask questions. My questions have been answered well enough. I had enough time to decide if I'm going to participate.
- I know that participation is voluntary. I also know that I can decide at any time not to participate in the study. Or to stop. I don't have to say why I want to stop.
- I give the investigator permission to let my GP know that I am participating in this study.
- I give the investigator permission to request information from my GP about my medical history and medication use.
- I give the investigator permission to give my GP or specialist information about unexpected findings from the study that are important for my health.
- I give the researchers permission to collect and use my data. The investigators are only doing this to answer the research question of this study.
- I know that for the purpose of checking the research, some people will be able to see all my data. Those people are listed in this information letter. I give these people permission to view my data for this check.
- I give the researcher permission to collect the following special personal data: my ethnicity.
- Would you like to tick yes or no in the table below?

| I give permission for my data to be stored in order to use it for other research on lifestyle and brain health, as stated in the information letter. | Yes ☐ | No☐ |
| --- | --- | --- |
| I give the researchers permission to let me know which version of the app I used after the study. | Yes ☐ | No☐ |
| If I decide to stop participating in the study early, the investigator may call me at the end of the study to ask why I stopped. | Yes ☐ | No☐ |
| If I have stopped the study, and am not available to answer questions about my health, I give the investigator permission to contact my GP or treating specialist. | Yes ☐ | No☐ |
| I give permission to keep my contact details so that the investigators can send me the most important results after the study has been completed. | Yes ☐ | No☐ |

I want to participate in this study.

My name is (participant): ......................................

Signature:........................... Date : __ / __ / __

-----------------------------------------------------------------------------------------------------------------

I declare that I have fully informed this subject about the said study.

Will any information become known during the study that may affect the subject's consent? Then I will let this test subject know in time.

Name of investigator (or his/her representative):.....................................

Signature:........................... Date:__/__/__

-----------------------------------------------------------------------------------------------------------------

*The subject will be provided with a full information letter, along with a signed version of the consent form.*

**Appendix 3a: Topic list for the intervention participants**

***This topic list may be updated based on information from the platform, questionnaires, or interviews with the coaches.***

**Disclaimer: This is a not a certified translated version of the original Dutch Interview guide. All interviews will be conducted in Dutch**

### Introduction

- Why did you decide to participate?
- What is your opinion on the intervention as a whole? What are its strengths, and what can be improved?
- Do you find the intervention as a whole appealing?
- Does the intervention meet your need to work towards a healthier lifestyle?
- Does the intervention fit well into your life?

### App

- What is your opinion of the app? What are its strengths, and what can be improved?
- Do you find the application user-friendly?
- Were there any practical barriers to using the app? For example, did you encounter login problems or missed reminders? If so, which ones?
- Are there other (social) barriers to using the app? If so, which ones?
- Is it enjoyable to use the app?
- Did you set goals at the beginning?
  - What goal(s)?
  - Why those goal(s)?
- Did the app help you set the goals in the way you wanted? (in terms of the type of goal, how to achieve it, how to measure it, etc.)
- Did you set new goals during the study? If so, how did that go? If not, what barriers did you encounter?
- Did you track your progress? For what? If so, how did that go? If not, what barriers did you encounter?
- Did you use the advice and education section?
- What do you think of the advice or explanations in the educational part of the app? Are they useful? Did you miss certain things?

### Coaching

- Did you communicate with the coach via chat? How did that go? Did it work well?
- Did you trust that the information you sent to your coach was received by them?
- What is your opinion of the coach?
- Did the coach's suggestions meet your needs? If not, how could that be improved?
- Is the combination of app use and coaching useful for you? Or would one of the two be sufficient?

### Intervention

You have now been participating for about six months.

- How did you find participating in the study over a longer period?
- Can you describe if you used the app more frequently during certain periods?
- What kept you going?
- Did you consider quitting? (Why?)
- What role did people around you play in the intervention? Did they encourage you to continue with the intervention?
- Did you visit your GP or other healthcare professionals because of the study? For instance, in relation to lifestyle, high blood pressure, or high cholesterol?
- Have you changed your lifestyle as a result of the intervention? If so, in what way? If not, what were the barriers?
- Were there practical barriers to changing your lifestyle? If so, which ones?
- Were there social barriers to changing your lifestyle? If so, which ones?

### Conclusion

- Did the MIND-PRO intervention help you achieve your goals?
- Compared to your expectations at the start of the study, did things go as you expected in terms of achieving your goals?

**Appendix 3b: Topic list for the coaches**

***This topic list may be updated based on information from the platform, questionnaires, or interviews with the intervention participants.***

**Disclaimer: This is a not a certified translated version of the original Dutch Interview guide. All interviews will be conducted in Dutch**

### Overall Intervention

- What is your opinion of the intervention as a whole? What are its strengths, and what can be improved?
- What is your general impression of how participants maintain the intervention?
- Do you feel there are specific characteristics among participants who drop out/do not participate in the study? If so, what were they? Were there participants more likely to engage effectively?
- Do you have suggestions to improve participant engagement?

### App

- Which app features were well or poorly used (and why)? Does this change as participants are involved in the study for longer or shorter durations?

### Coaching

- Can you tell me about your experiences coaching people during the study?
- Can you give me examples of participants who seemed successful? How did you (attempt to) coach them, and what did you learn to improve coaching?
- Were you able to apply motivational interviewing techniques? Can you provide an example?
- Can you provide examples of coaching techniques that worked well and those that didn’t work well?
- What is your opinion on remote coaching over a year? Is it easy/difficult? Why?
- Can you provide examples of participants for whom the MIND-PRO intervention worked reasonably well remotely and for whom more personal contact (e.g., phone calls or face-to-face contact) was needed? [How did they differ in terms of personality, type of goal, or otherwise?]

### Coach Portal

- What did you think of the online coach portal?
- Did you miss anything in the coach portal?
- Did you use advice or explanations in the educational section of the app in your coaching? Why/why not? Do you think there are important topics missing in the educational section?

### Coach Meetings

- What did you think of the scheduled meetings to discuss coaching? Has this changed over the course of the study?
- Which aspects of these meetings were valuable for you? (Can you provide examples?) Do you have suggestions to improve these meetings?

### Future Use

We would like to improve the intervention for future use.

- What advice would you give us about modifications or redesign?
- Should it be offered as part of preventive healthcare in primary care?
- What is needed to implement the intervention in regular healthcare?
