# Supplementary material for: mHealth Intervention for Dementia Prevention through lifestyle Optimisation (MIND-PRO) in a primary care setting: protocol for a randomised controlled trial in people with low SES and/or migration background
Source: BMJ Open. 2025 Feb 3;15(2):e088324. doi: 10.1136/bmjopen-2024-088324 (PMC11956295; doi:10.1136/bmjopen-2024-088324)
Supplement: Supplementary file 2 [file bmjopen-2024-088324.reviewer_comments.pdf]

PEER REVIEW HISTORY

BMJ Open publishes all reviews undertaken for accepted manuscripts. Reviewers are asked to complete a checklist review form and are provided with free text boxes to elaborate on their assessment. These free text comments are reproduced below.

ARTICLE DETAILS

Title (Provisional)

mHealth Intervention for Dementia Prevention through lifestyle Optimisation (MIND-PRO) in a primary care setting: protocol for a randomized controlled trial in people with low SES and/or migration background

Authors

van der Endt , Anne Roos; Hoevenaar-Blom, Marieke P; Galenkamp, Henrike; Kas, Martien J.H.; van den Berg, Esther; Handels, Ron; Moll van Charante, Eric P; Richard, Edo

---

VERSION 1 - REVIEW

**Reviewer**	**1**
**Name**	**Whitehouse, Peter**
**Affiliation**	**Case Western Reserve University**
**Date**	**08-Aug-2024**
**COI**	**I have co-authored a paper with one of the current authors, Edo Richard.**

This paper described a 12-month protocol for a type2 hybrid effectiveness-implementation trial designed to reduce dementia risk in a target population of low SES and immigrants to The Netherlands. It has biomarkers as primary measures and dementia risk scores as secondary. The two arms are a human coach-supported mobile phone app and a static information control.

The design is well outlined. The recruitment, screening, and randomization processes are adequately described as are the power calculations. One wonders whether that static arm should include some opportunity for asking questions of humans. That would control for the possibility that the intervention is due just to human contact at and not to dynamic personalized information. That might allow better blinding as well. The authors point out that the risk factors and markers are relevant to cardiovascular disease, not just dementia. How can they try to sort out benefit to heart and brain? They do not necessarily have to care for their outcome measures, but it would be interesting to try. Are they informing the

subject that the intervention may help their cardiovascular health as well as brain health? It might provide greater motivation for some? Will they have access to date about frequency of use of the app and contact with coaches? It would be interesting to see if a dose-response curve could be developed to see if more frequent and longer engagement with the intervention leads to better outcomes. Post intervention debriefings and exploration of unblinding episodes could be important in understanding how to improve future designs. The processes could be better described as could how the authors engage the target populations in the original design of the study.

Reviewer **2**

Name **Pond, Dimity**

Affiliation **University of Newcastle Australia, General Practice**

Date **09-Aug-2024**

COI **I am a member of a research group conducting a telephone intervention similar to this one.**

Thank you for this interesting paper, addressing an important question regarding dementia risk in disadvantaged populations.

My main comments relate to the handling of the risk factor depression in the manuscript. This I believe is a little unclear from the beginning.

IN the abstract depression is appropriately mentioned as a risk factor and is included as a secondary outcome.

Depression is not mentioned as one of the risk factors addressed through the App under the heading "Intervention and control condition" although it is mentioned as a component of the secondary effectiveness outcomes. It is unclear to this reviewer how the app education and/or coaches would address depression. Is it perhaps thought to improve in line with the primary outcomes? If so, this should be mentioned and the rationale explained (eg is it thought to be linked to physical activity/ cognitive function improvement?).

I note that in the data analysis section, the scales for depressive symptoms are mentioned in a group with those for disability and cognitive function. Although the scales for cognitive function are mentioned, one must search the appendices to find those measuring depression and disability. However, this is a minor point.

I am not sufficiently skilled in statistics to comment in detail on the proposed analysis.

Reviewer **3**

Name **Ma, Kris Pui Kwan**

Affiliation University of Washington

Date 03-Nov-2024

COI N/A

This is a well-written study protocol that describes a randomized controlled trial testing an app-based lifestyle modification intervention for preventing dementia in underserved populations – people with low SES and/or migration background.

There are several questions that this reviewer believes the protocol should clarify:

1. Provide rationale and literature support for choosing another app as a control condition

- Since participants in both conditions will be using an app: one with interactions with health coach and self-monitoring, and the other one is an educational app with evidence-based education modules on healthy lifestyles, there seems to be a premise underlying this study that assumes there will be differences in intervention effects on health outcomes between an interactive app (with features such as coaching and self-monitoring that facilitate behavioral change), as compared to a passive educational app. Hence, it is important to provide literature background to support this premise and hypothesis (if this is the hypothesis for this study).

- Will the app in the control condition have cultural-specific content and be available in different languages?

2. What would be some way to assess and account for systematic differences (if any) between participants recruited from HELIUS study vs. from other primary care practices?

- There is a possibility that different recruitment sources can recruit various participants. While randomization can potentially help reduce selection bias and confounders, this reviewer suggests assessing for potential differences at baseline between these two groups of participants first before randomization. This information will also be helpful to understand how different recruitment and outreach methods can yield different groups of participants within populations of low SES and/or migration background.

3. Could the authors provide more information about the training and credentials of the coaches?

- What are the credentials or specialty training for these coaches?

- How much training and experience do they have in lifestyle modification/health behavior changes, in addition to motivational interviewing?

- What are the roles and 'scope of practice' of these health coaches? (things that they do and things that are outside of their scope)

4. Do the authors also plan to collect data on participants' medications use and primary care visits/health care utilization during the 12-month intervention period? Since these could potentially be confounding variables.

VERSION 1 - AUTHOR RESPONSE

Reviewer: 1
Dr. Peter Whitehouse, Case Western Reserve University
Comments to the Author:
This paper described a 12-month protocol for a type2 hybrid effectiveness-implementation trial designed to reduce dementia risk in a target population of low SES and immigrants to The Netherlands. It has biomarkers as primary measures and dementia risk scores as secondary. The two arms are a human coach-supported mobile phone app and a static information control.

The design is well outlined. The recruitment, screening, and randomization processes are adequately described as are the power calculations. One wonders whether that static arm should include some opportunity for asking questions of humans. That would control for the possibility that the intervention is due just to human contact at and not to dynamic personalized information. That might allow better blinding as well.
Thank you for you remark about adding human interaction to the control arm. We understand this suggestion. Part of our research aim however, is to investigate whether a blended intervention (app + remote coach) is effective in reducing dementia risk factors. This is why we did not add human interaction to the control condition. The reviewer is correct that it will not be possible to determine which aspect of the blended intervention contributed most to any effect of the intervention from a quantitative perspective. In interviews we will explore this qualitatively.

The authors point out that the risk factors and markers are relevant to cardiovascular disease, not just dementia. How can they try to sort out benefit to heart and brain? They do not necessarily have to care for their outcome measures, but it would be interesting to try.
The reviewer is correct in stating that our intervention targets dementia risk factors, which largely overlap with those for cardiovascular disease. As this is a proof-of-concept study with a 12-months intervention period we do not expect to see any effect on incident dementia or cardiovascular disease. For this, future large-scale randomized controlled trials with a longer follow-up period are needed, if our proof-of-concept study is beneficial on dementia risk factors.

Are they informing the subject that the intervention may help their cardiovascular health as well as brain health? It might provide greater motivation for some?
Thank you for this comment, we agree that informing participants about the potential effects for the heart and brain of our intervention might enhance motivation to participate. In our Patient Information Form (appendix 3) we inform participants about the benefits of a healthy lifestyle for their heart in addition to their brain, as this is a more widely understood concept. Research in the Netherlands has shown that many people are unaware of the relationship

between lifestyle and dementia.(1) By emphasizing the potential benefits for brain health in addition to cardiovascular health, we aimed to enhance participants' motivation to engage with the study without causing anxiety about dementia, as this connection might be unfamiliar and potentially distressing for some.

Will they have access to date about frequency of use of the app and contact with coaches? It would be interesting to see if a dose-response curve could be developed to see if more frequent and longer engagement with the intervention leads to better outcomes.
Thank you for this suggestion. We completely agree that this would be an interesting are to investigate. At the time of writing our protocol, we were uncertain whether it would be possible to measure the frequency of app use and contact with coaches. However, we now know that it is possible to measure the number of log-in to the app (i.e. how many times participants opened the app) and number of chat messages sent to the coaches. We will perform an additional analysis to investigate a potential dose-response relationship.
We added this to line 256-257 in the statistical analysis part of the manuscript.

Post intervention debriefings and exploration of unblinding episodes could be important in understanding how to improve future designs.
Thank you for the interesting remark. We agree that this would be relevant in a study where participants are aware of the existence of an active app and a sham app. However, we only inform our participants that there are two versions of the app without explaining the difference. Therefore, they are not aware that one of the two arms is more active than the other.
We clarified this in the manuscript, lines 136-139

The processes could be better described as could how the authors engage the target populations in the original design of the study.
Thank you for pointing this out, we adjusted the text in our manuscript in lines 141-148.

Reviewer: 2
Dr. Dimity Pond, University of Newcastle Australia
Comments to the Author:
Thank you for this interesting paper, addressing an important question regarding dementia risk in disadvantaged populations.
My main comments relate to the handling of the risk factor depression in the manuscript. This I believe is a little unclear from the beginning.
IN the abstract depression is appropriately mentioned as a risk factor and is included as a secondary outcome.
Depression is not mentioned as one of the risk factors addressed through the App under the heading "Intervention and control condition" although it is mentioned as a component of the secondary effectiveness outcomes. It is unclear to this reviewer how the app education and/or coaches would address depression. Is it perhaps thought to improve in line with the primary outcomes? If so, this should be mentioned and the rationale explained (eg is it thought to be linked to physical activity/ cognitive function improvement?).
I note that in the data analysis section, the scales for depressive symptoms are mentioned in a group with those for disability and cognitive function. Although the scales for cognitive function are mentioned, one must search the appendices to find those measuring depression and disability.

However, this is a minor point.
I am not sufficiently skilled in statistics to comment in detail on the proposed analysis.

Thank you for raising the point about depression as a risk factor in our trial. To select a high-risk population for our study, we pre-defined several known modifiable risk factors for dementia, including depression. However, while depression is used as a criterion for selecting participants, it is not a specific focus of our intervention. Our coaches are trained to support participants in making lifestyle changes but are not equipped to address mental health issues, such as depression, which require specialized training and a different approach. It is however possible that depressive symptoms may be indirectly influenced by improvements in self-management of health and physical activity. These improvements in their mood will be measured through the GDS-15, reported as a secondary outcome in this study.

To clarify this, we added lines 158-160 to the manuscript.

Reviewer: 3
Dr. Kris Pui Kwan Ma, University of Washington
Comments to the Author:
This is a well-written study protocol that describes a randomized controlled trial testing an app-based lifestyle modification intervention for preventing dementia in underserved populations – people with low SES and/or migration background.

There are several questions that this reviewer believes the protocol should clarify:
1. Provide rationale and literature support for choosing another app as a control condition
- Since participants in both conditions will be using an app: one with interactions with health coach and self-monitoring, and the other one is an educational app with evidence-based education modules on healthy lifestyles, there seems to be a premise underlying this study that assumes there will be differences in intervention effects on health outcomes between an interactive app (with features such as coaching and self-monitoring that facilitate behavioral change), as compared to a passive educational app. Hence, it is important to provide literature background to support this premise and hypothesis (if this is the hypothesis for this study).
- Will the app in the control condition have cultural-specific content and be available in different languages?

Thank you for your comments and for reviewing our study protocol. We agree with the reviewer that it cannot be ruled out that there might be an effect in the control condition by only using the static library with educational materials. We would like to clarify that these are no educational modules, but consist of plain information on a healthy lifestyle only. We do not expect this information will lead to a major health benefit, based on extensive literature indicating that merely promoting health knowledge is ineffective when it comes to sustainable behavior change.(2) Building on this, the literature shows that features such as (remote) coaching and self-monitoring of health could contribute to behavior change and are thus included the intervention arm.(3-6).

The app used in the control condition will not include culturally specific content and will be available in Dutch and Turkish.

This was added to the manuscript lines: 138-139

2. What would be some way to assess and account for systematic differences (if any) between participants recruited from HELIUS study vs. from other primary care practices?
- There is a possibility that different recruitment sources can recruit various participants. While randomization can potentially help reduce selection bias and confounders, this reviewer suggests assessing for potential differences at baseline between these two groups of participants first before randomization. This information will also be helpful to understand how different recruitment and outreach methods can yield different groups of participants within populations of low SES and/or migration background.

Coverage compared to the source population is indeed an important outcome. Coverage will be measured as one of our primary implementation outcomes.
To gain insight to which degree the people participating in the MIND PRO study represent the source population, comparisons will be made between study participants at baseline and participants of the HELIUS population aged 50-75 with at least one risk factor.

As we recruit participants of low SES and/or migration background, inherent differences will exist compared to individuals from other primary care practices. Consequently, comparing comparing characteristics between these groups is not informative. Among all invited participants for the HELIUS study, 28% chose to participate. The reviewer is correct in noting that recruiting from an existing cohort potentially reduces the external validity of the study with respect to the broader target population. However, this approach enables focused recruitment and ensures representation from the groups most relevant to our research question.

3. Could the authors provide more information about the training and credentials of the coaches?
- What are the credentials or specialty training for these coaches?
- How much training and experience do they have in lifestyle modification/health behavior changes, in addition to motivational interviewing?
- What are the roles and 'scope of practice' of these health coaches? (things that they do and things that are outside of their scope)

Thank you for raising this question. All coaches have some experience in (lifestyle) coaching, with one head-coach with extensive experience in both her own practice and training other coaches. All are all trained in motivational interviewing techniques.

Added in de the manuscript line 118-119

The coaches play a key role in guiding participants through their lifestyle changes. They are advised to direct participants with medical questions to consult their doctor. Similarly, questions related to mental health fall outside the scope of this intervention and will prompt a recommendation to seek assistance from a healthcare professional.

> *Added in the manuscript line 122-125*

4. Do the authors also plan to collect data on participants' medications use and primary care visits/health care utilization during the 12-month intervention period? Since these could potentially be confounding variables.

We agree that this is important information. We collect data on the use of antihypertensive medications, cholesterol-lowering drugs, diabetes medications, and antidepressants at both the start and end of the study. Additionally, at six months post-randomization and again at the end of the intervention, participants are asked about their healthcare utilization. Specifically, we ask whether they visited a general practitioner (GP), physiotherapist, or dietician, as well as the frequency of these visits. We also collect information on any hospital visits during the same period. This information on healthcare utilization will be used for an economic evaluation.

This was added to the methods section in lines 183-184 (medication use), 190-191 (healthcare utilization), and 197.

1. Heger I, Deckers K, van Boxtel M, de Vugt M, Hajema K, Verhey F, Köhler S. Dementia awareness and risk perception in middle-aged and older individuals: baseline results of the MijnBreincoach survey on the association between lifestyle and brain health. BMC public health. 2019;19:1-9.
2. Webb T, Joseph J, Yardley L, Michie S. Using the internet to promote health behavior change: a systematic review and meta-analysis of the impact of theoretical basis, use of behavior change techniques, and mode of delivery on efficacy. Journal of medical Internet research. 2010;12(1):e1376.
3. Jakob R, Harperink S, Rudolf AM, Fleisch E, Haug S, Mair JL, et al. Factors Influencing Adherence to mHealth Apps for Prevention or Management of Noncommunicable Diseases: Systematic Review. J Med Internet Res. 2022;24(5):e35371.
4. Shabir H, D'Costa M, Mohiaddin Z, Moti Z, Rashid H, Sadowska D, et al. The Barriers and Facilitators to the Use of Lifestyle Apps: A Systematic Review of Qualitative Studies. European Journal of Investigation in Health, Psychology and Education. 2022;12(2):144-65.
5. Yang M, Duan Y, Liang W, Peiris D, Baker JS. Effects of face-to-face and ehealth blended interventions on physical activity, diet, and weight-related outcomes among adults: a systematic review and meta-analysis. International Journal of Environmental Research and Public Health. 2023;20(2):1560.
6. Jongstra S, Beishuizen C, Andrieu S, Barbera M, van Dorp M, van de Groep B, et al. Development and validation of an interactive internet platform for older people: the healthy ageing through internet counselling in the elderly study. Telemedicine and e-Health. 2017;23(2):96-104.
